# Enantioselective C–C Bond Formation as a Result of the Oriented Prochirality of an Achiral Aldehyde at the Single-Crystal Face upon Treatment with a Dialkyl Zinc Vapor**

**DOI:** 10.1002/anie.201102031

**Published:** 2011-06-15

**Authors:** Tsuneomi Kawasaki, Sayaka Kamimura, Ai Amihara, Kenta Suzuki, Kenso Soai

**Affiliations:** Department of Applied Chemistry and Research Institute for Science and Technology, Tokyo University of ScienceKagurazaka, Shinjuku-ku, Tokyo 162-8601 (Japan)

**Keywords:** achiral crystal lattice, enantioselectivity, enantiotopic faces, prochiral aldehydes, zinc

The origin of biological homochirality, such as that seen in l amino acids and d sugars, is one of the most important subjects for broad research.[1] Circularly polarized light,[2] chiral inorganic crystals,[3] such as quartz,[3c] chiral organic crystals,[4] and spontaneous absolute asymmetric synthesis[5] have been proposed as candidates for the origin of chirality. Supramolecular arrangement by an external chiral factor has also been suggested.[6] The induced chirality should be enhanced to high enantiomeric enrichment by suitable multiplication and amplification mechanisms,[7,8] such as amino acid catalyzed aldol reactions[9] and asymmetric autocatalysis.[10]

Lahav,[4e] Holland and Richardson[11] originally suggested the concept of a reaction at the enantiotopic face of an achiral single crystal[11a] and later reported oxidation reactions of olefinic compounds.[11b] Because the reagents reacted directly with the oriented molecules in the crystal, the products formed in a stereospecific manner to provide optically active compounds corresponding to the prochirality of the substrate at the crystal surface. Since chiral compounds can be obtained from achiral compounds,[12] enantioselective reactions on a selected face have been considered as another possible origin of chirality. Recently, Kuhn and Fischer reported a reduction at the enantiotopic surface of a ketone to provide a chiral alcohol with up to 26 % *ee*.[13] Some chiral effects at enantiotopic surfaces have been reported, such as molecular recognition,[14a] crystallization,[14b] and dehydration.[14c]

Thus, enantioselective C–C bond formation at specific enantiotopic surfaces is a challenge. We herein report the enantioselective addition of diisopropylzinc (*i*Pr_2_Zn) at a particular single-crystal face of aldehyde **1** to form a chiral secondary alcohol **2** (Scheme 1). When a single-crystal surface was treated with *i*Pr_2_Zn vapor, the enantioselective isopropylation proceeded to afford the chiral 5-pyrimidyl alkanol **2** with the absolute configuration corresponding to the oriented prochirality of the achiral aldehyde **1**.

**Scheme 1:**
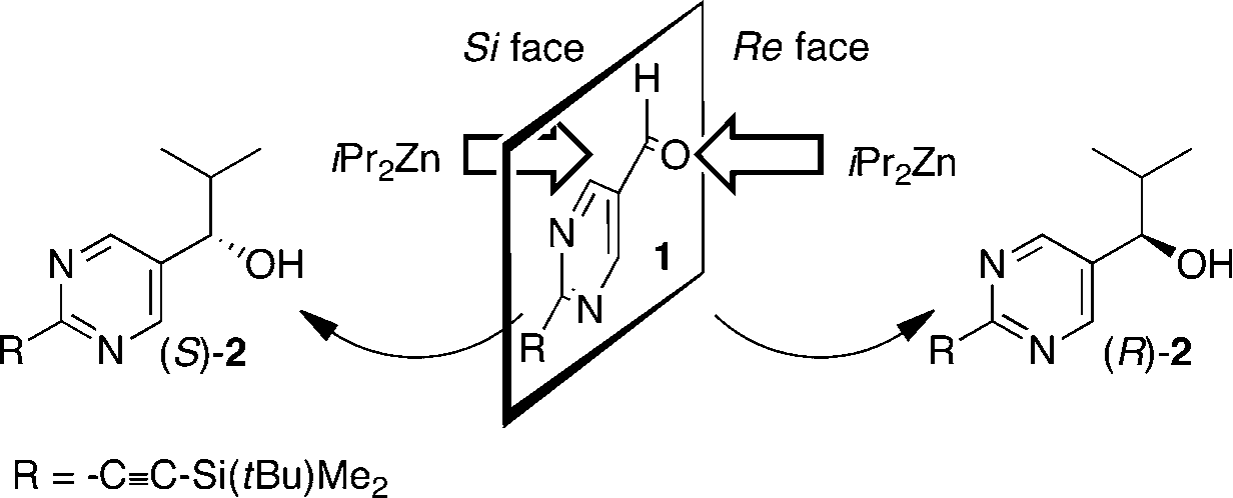
Enantioselective addition of diisopropylzinc to the pyrimidine-5-carbaldehyde **1** to form the 5-pyrimidyl alkanol **2**.

We previously reported that 2-(alkylethynyl)- and 2-(trialkylsilylethynyl)pyrimidine-5-carbaldehyde[15] serve as excellent substrates in asymmetric autocatalysis with the amplification of enantiomeric excess.[16] Thus, as an achiral substrate, we selected 2-(*tert*-butyldimethylsilylethynyl)pyrimidine-5-carbaldehyde (**1**), which can be prepared from 5-bromo-2-iodopyrimidine by a coupling reaction with *tert*-butyldimethylsilylacetylene and formylation (see the Supporting Information). A single crystal of **1** with well-defined crystal faces could be obtained by recrystallization from a solvent mixture of cumene and ethyl acetate by slow evaporation (Figure 1a). Single-crystal X-ray structure analysis demonstrated that aldehyde **1** belongs to the achiral space group 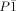, and the large parallelogram surfaces were determined to be enantiotopic (001) and (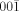) faces (Figure 1b).[17] These faces are colored sky blue and yellow in the unit-cell structure (Figure 1c). When aldehyde **1** was projected onto the yellow-colored face, the *Si* face of its formyl group was oriented toward the outside of the crystal; thus, the *Re* face was oriented toward the opposite blue-colored face.

**Figure 1:**
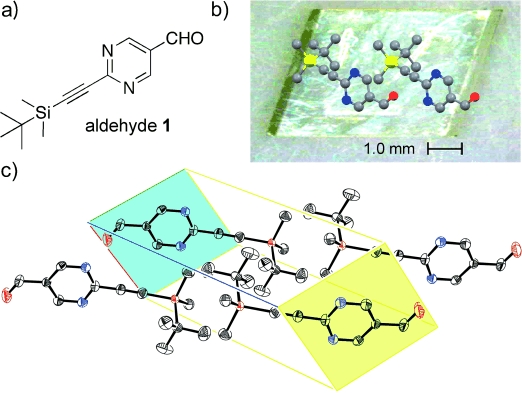
a) Structure of aldehyde **1** (space group: 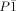). b) Microscopic image of the single crystal **1** and relative orientation of the prochiral aldehyde **1** at the (001) face. c) Unit cell of crystal **1**. The yellow and blue planes correspond to enantiotopic surfaces.

For the enantioface-selective addition of *i*Pr_2_Zn, the single crystal, apart from the single reactive surface, was coated with an epoxy resin (Figure 2a), so that *i*Pr_2_Zn vapor could access only one enantiotopic face. The enantiotopic (001) and (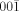) faces were defined on the basis of the parallelogram face shape and were independently exposed to *i*Pr_2_Zn vapor without the use of a solvent for the reaction (Figure 2b). Dissolution would cause the disappearance of the molecular orientation of the achiral aldehyde in crystal **1**.

**Figure 2:**
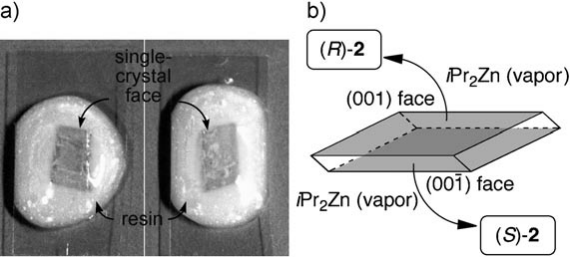
Enantioselective addition of diisopropylzinc to aldehyde **1** at an enantiotopic face of the single crystal **1**. a) Apart from the single reactive (enantiotopic) surface, crystal **1** was coated with an epoxy resin. b) Enantiotopic parallelogram (001) and (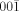) faces were exposed to diisopropylzinc vapor independently.

When the enantiotopic (001) face was exposed to *i*Pr_2_Zn for the addition reaction, alkanol (*R*)-**2** (2.7 mg) was isolated with 46 % *ee* in 19 % yield based on the weight of the single crystal **1** (#1; Table 1, entry 1). The reaction at the morphologically determined (001) face afforded (*R*)-**2** with 50–67 % *ee* and excellent reproducibility (Table 1, entries 2–4). On the other hand, when the (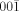) face was exposed to *i*Pr_2_Zn, the opposite enantiomer (*S*)-**2** was produced with 14–62 % *ee* (Table 1, entries 5–8).

**Table 1:** Correlation between the exposed enantiotopic crystal face of aldehyde **1** and the absolute configuration of the alcohol product **2**.[a]

Entry	Single crystal		Reactive face		**2**		
	no.	weight	face	area	yield[b]		*ee*[c] [%]
		[mg]	index	[mm^2^]	[mg]	[%]	(config.)
1	#1	12	(001)	20	2.7	19	46 (*R*)
2	#2	n.d.[d]	(001)	n.d.[d]	1.6	n.d.[d]	56 (*R*)
3	#3	n.d.[d]	(001)	n.d.[d]	1.0	n.d.[d]	50 (*R*)
4	#4	n.d.[d]	(001)	n.d.[d]	0.9	n.d.[d]	67 (*R*)
5	#5	12	( 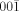 )	20	2.5	18	62 (*S*)
6	#6	5	( 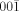 )	8	1.7	29	14 (*S*)
7	#7	n.d.[d]	( 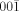 )	n.d.[d]	1.3	n.d.[d]	30 (*S*)
8	#8	n.d.[d]	( 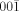 )	n.d.[d]	2.2	n.d.[d]	22 (*S*)
9	#9	6	(001)	7	4.4	62	55 (*R*)
10	#9	6	( 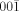 )	7	5.6	80	48 (*S*)
11	#10	15	(001)	14	5.1	29	31 (*R*)
12	#10	15	( 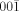 )	14	4.9	28	69 (*S*)
13	#11	18	(001)	23	5.7	27	43 (*R*)
14	#11	18	( 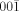 )	23	5.3	25	15 (*S*)
15	#12	8	(001)	15	2.0	21	45 (*R*)
16	#12	8	( 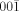 )	15	2.2	23	36 (*S*)
17[e]	#13	12	(001)	20	2.2	78	>99.5 (*R*)
18[e]	#13	12	( 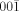 )	20	1.8	75	>99.5 (*S*)

[a] The addition reaction was performed in a 50 mL flask filled with *i*Pr_2_Zn vapor (see Figure S1).

[b] Yield of isolated **2** without regard to unreacted aldehyde **1**. After the reaction had been quenched, a TLC experiment showed the presence of only the product **2** and unreacted **1**.

[c] The *ee* value was determined by HPLC on a chiral stationary phase (see Figure S2).

[d] Not determined.

[e] Asymmetric autocatalysis with amplification of the *ee* value was performed with alcohol **2** obtained from the solid–gas reaction (see Table S1). Before the amplification of enantiomeric excess, the *ee* values for the products in entries 17 and 18 were 44 and 31 %, respectively.

As the relationship between the absolute configuration of product **2** and the parallelogram face shape of reactant **1** was reproducibly constant, the orientation of prochiral aldehyde **1** in the crystal should correlate to the crystal morphology. Aldehyde **1** was not completely consumed in these solid–gas reactions; therefore, low chemical yields were observed. The wide variety of *ee* values should be due to the quality of the single crystal used as the reactant.

To make sure of the stereochemical relationship, we conducted the exposure experiments by using opposite enantiotopic faces originating from one specific single crystal, which was cut into two pieces (Table 1, entries 9–16). In the reaction in entry 9 of Table 1, the (001) face of one half of the crystal (#9) was exposed to *i*Pr_2_Zn vapor to afford (*R*)-**2** with 55 % *ee* in 62 % yield. In contrast, reaction at the (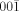) face afforded (*S*)-**2** with 48 % *ee* (Table 1, entry 10). The reproducibility of the formation of the major enantiomer was demonstrated clearly with single crystals #10–#12 (Table 1, entries 11–16).

Alcohol **2** can also act as a highly efficient asymmetric autocatalyst in the homogeneous solution state.[10] Therefore, the obtained alcohol **2** was subjected to asymmetric autocatalysis with amplification of enantiomeric enrichment;[16b] this process afforded almost enantiomerically pure (*R*)- and (*S*)-**2** with more than 99.5 % *ee* (Table 1, entries 17 and 18; see also Table S1 in the Supporting Information).

We believe that the enantioselectivity observed in the present reaction is induced by the direct reaction of *i*Pr_2_Zn vapor at a particular crystal face at which either the *Si* or the *Re* enantioface of the aldehyde is aligned with the outside of the crystal. By the use of one specific surface for the reaction, the direction of the nucleophile approach to the aldehyde **1** can be controlled. Therefore, chiral induction is possible through the choice of one enantiotopic face of an achiral single crystal **1**. The formation of a racemate would be expected if the reaction occurred at both enantiotopic surfaces of a crystal, neither of which had been coated with a resin.

In summary, we have demonstrated the enantioface-selective addition of *i*Pr_2_Zn to pyrimidine-5-carbaldehyde **1**. By selecting one enantiotopic crystal face, the chiral secondary alcohol **2** was formed with the absolute configuration corresponding to the two-dimensional chirality at the crystal surface. We could predict the absolute configuration of alcohol **2** from the parallelogram face shape. Furthermore, the *ee* value of product **2** could be enhanced to greater than 99.5 % by asymmetric autocatalysis with amplification of enantiomeric enrichment.

## Experimental Section

Crystals of aldehyde **1** were grown from a solution of **1** in cumene and ethyl acetate (3:1, v/v) by slow evaporation at room temperature for 1–2 days. A single crystal of **1** was coated with quick-set epoxy glue (Araldite), except for the reactive enantiotopic surface; for this purpose, the crystal was placed with this face against a glass slide. The crystal with one enantiotopic face exposed was put into a two-necked 50 mL flask. A 1 m solution of *i*Pr_2_Zn in cumene (1 mL) was placed in another vessel, which was fitted to the 50 mL flask (see Figure S1 in the Supporting Information). The crystal was exposed to *i*Pr_2_Zn vapor for 24 h at room temperature. Although the crystal turned yellow, the crystal shape remained unchanged, without dissolution or the formation of a suspension. The reaction was quenched with water-saturated ethyl acetate. The organic layer was washed with water and dried over anhydrous sodium sulfate. After evaporation in vacuo, the remaining residue was purified by silica-gel column chromatography with hexane/ethyl acetate (3:1, v/v) as the eluent to give **2** as a colorless solid. The *ee* value was determined by HPLC on a chiral stationary phase.

Data for **1**: colorless crystal; m.p.: 116.6–117.0 °C (cumene and ethyl acetate); IR (KBr): 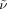
=2952, 2928, 2854, 1709, 1576, 1542, 1416, 1205, 866, 830, 782 cm^−1^; ^1^H NMR (600 MHz, CDCl_3_): *δ*=0.257 (6 H, s), 1.03 (9 H, s), 9.14 (2 H, s), 10.14 ppm (1 H, s); HRMS: *m*/*z* calcd for C_13_H_18_N_2_SiONa^+^: 269.1081 [*M*+Na]^+^; found: 269.1076.
