# Supplementary material for: Enantioselective C–C Bond Formation as a Result of the Oriented Prochirality of an Achiral Aldehyde at the Single-Crystal Face upon Treatment with a Dialkyl Zinc Vapor
Source: Angew Chem Int Ed Engl. 2011 Jun 15;50(30):6796–8. doi: 10.1002/anie.201102031 (PMC3170706; doi:10.1002/anie.201102031)
Supplement: Supplementary file 1 [file anie0050-6796-SD1.pdf]

Angewandte Chemie
Eine Zeitschrift der Gesellschaft Deutscher Chemiker

Supporting Information

© Wiley-VCH 2011

69451 Weinheim, Germany

Enantioselective C–C Bond Formation as a Result of the Oriented Prochirality of an Achiral Aldehyde at the Single-Crystal Face upon Treatment with a Dialkyl Zinc Vapor**

Tsuneomi Kawasaki, Sayaka Kamimura, Ai Amihara, Kenta Suzuki, and Kenso Soai**

anie_201102031_sm_miscellaneous_information.pdf

1. General comments:

All reactions were carried out under argon atmosphere. Reactions and purifications were monitored by thin layer chromatography using Silica gel 60 F_{254} (pre-coated on aluminum sheet, 0.2 mm thickness, Merck). Chromatographic purification was performed with Silica gel 60 (230–400 mesh, Merck). Diffraction data were obtained on a Rigaku, SCXmini using a graphite monochromated Mo Kα radiation. Infrared (IR) data were recorded on a Jasco FT-IR-4100ST spectrometer as thin film using sodium chloride plates for oily compounds, as KBr disk or nujol mulls for crystalline compounds. Optical rotations were measured using a Jasco P-1030 digital polarimeter using 5 cm cells. High-resolution mass spectra were recorded using a ESI-TOF mass spectrometers (Bruker Daltonics micrOTOF focus). NMR spectra were recorded on a BRUKER AV600 spectrometer (operating at 600 MHz for ^{1}H and 150 MHz for ^{13}C acquisitions). Chemical shifts d are reported in ppm with the solvent resonance as the internal standard (Chloroform-*d*: 7.26 (^{1}H-NMR), 77.0 (13C-NMR)). Coupling constants *J* are given in Hertz (Hz). Optical rotations were measured using a Jasco P-1030 digital polarimeter using 5 cm cells.

2. Experimental procedure for the syntheses of achiral aldehyde 1 and alkanol 2:

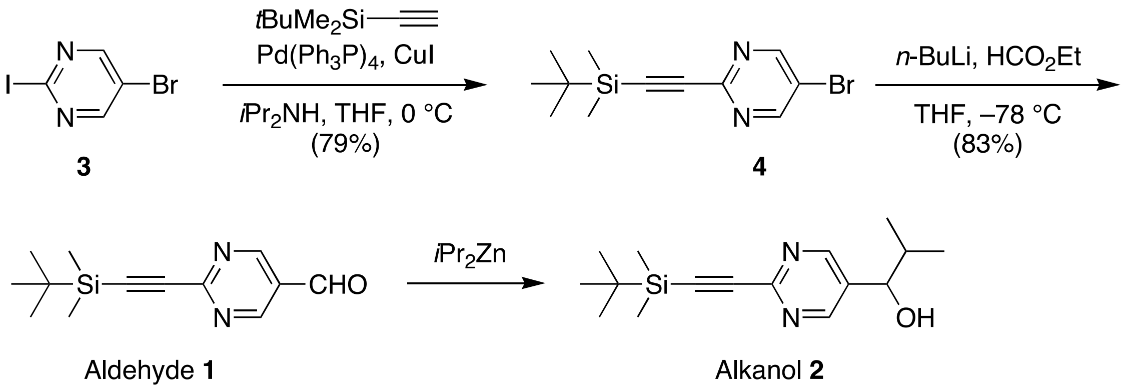

Scheme S1. Synthetic scheme of aldehyde **1** and alkanol **2** by *i*Pr_2Zn addition to **1**.

Compound 4: To a mixture of 5-bromo-2-iodopyrimidine **3** (2.85 g, 10 mmol), *tert*-butyldimethylsilylacetylene (2.0 mL, 10.7 mmol), *N,N'*-diisopropylamine (5.61 mL, 40 mmol), cupper iodide (38.1 mg, 0.2 mmol) and $(Ph_3P)_4Pd$ (116 mg, 0.1 mmol) was added under an argon atmosphere. After stirring for 12 h at 0 °C, the reaction mixture was filtered through Celite and the filtrate was concentrated in vacuo. The residue was purified by silica gel column chromatography using hexane:ethyl acetate (50:1, *v/v*) as an eluent to give product **4** (2.34 g, 7.78 mmol) in 79% yield.

Data for compound 4: Colorless solid; mp. 100.3–100.8 °C (hexane); ^{1}H-NMR (600 MHz, $CDCl_3$) δ (ppm) 0.231 (6H, s), 1.013 (9H, s), 8.754 (2H, s); ^{13}C-NMR (150 MHz, $CDCl_3$) δ (ppm) –4.74, 16.90, 26.32, 94.99, 102.44, 119.66, 150.49, 158.17; IR (Nujol) ν (cm^{-1}) 1523, 1407, 1253, 1112, 1008, 937, 842, 780, 702; HRMS Calcd. for $C_{12}H_{17}BrN_2SiNa^+$ $[M+Na]^+$: 319.0237, found. 319.0236.

Aldehyde 1: To a solution of 2-(*tert*-Buthyldimethylsilyl)ethenyl-5-bromopyrimidine (2.08 g, 7.0 mmol) in THF (70 mL), 1.67 M solution of *n*-butyllithium (4.19 mL, 7.0 mmol, hexane solution) was added dropwise at –78 °C . After the dropwise addition of ethyl formate (1.67 mL, 21 mmol), the reaction was quenched with 4N HCl in ethyl acetate (1.75 mL). The mixture was neutralized with saturated aqueous $NaHCO_3$ (5.25 mL) at room temperature. The mixture was extracted with EtOAc and the combined organic layer was dried over Na_2SO_4 and evaporated in vacuo. The remaining residue was purified by silica gel column chromatography (twice, eluent: hexane:ethyl acetate (3:1, v/v) and ether:hexane (5:1, v/v)) to give compound **1** (1.42 g, 5.78 mmol) in 83% yield. Single crystal was grown from the solution of aldehyde **1** in cumene and ethyl acetate (3/1, *v/v*) by the slow evaporation at room temperature for 1 to 2 days.

Data for aldehyde 1: Colorless crystal; mp. 116.6-117.0 °C (hexane); ^{1}H-NMR (600 MHz, $CDCl_3$) δ (ppm) 0.257 (6H, s), 1.03 (9H, s), 9.14 (2H, s), 10.14 (1H, s); ^{13}C-NMR (150 MHz, $CDCl_3$) δ (ppm) –4.80, 16.95, 26.32, 98.71, 103.05, 126.84, 155.59, 158.47,188.40; IR (KBr) ν (cm^{-1}) 2952, 2928, 2854, 1709, 1576, 1542, 1416, 1205, 866, 830, 782; HRMS Calcd. for $C_{13}H_{18}N_2SiONa^+$ [M+Na]$^+$: 269.1081, found: 269.1076.

Enantioselective addition of *i*Pr_2Zn to aldehyde 1: Single crystal **1** was coated with quick set epoxy glue (Araldite®) omitting the reactive enantiotopic surface on the slide glass. And the crystal, whose one enantiotopic face was exposed, was put into a two-necked 50 mL flask. And a 1 M solution of diisopropylzinc in cumene (1 mL) was added to another vessel, which was fitted to the 50 mL flask (Figure S1). The crystal was exposed to *i*Pr_2Zn vapor for 24 hours at room temperature. Although the color of the crystal was turned to be yellow, the crystal shape found to be remained without dissolution or suspension. The reaction was quenched with water-saturated ethyl acetate. The organic layer was washed with water and dried over anhydrous sodium sulfate. After the evaporation in vacuo, the remaining residue was purified by silica gel column chromatography using hexane:ethyl acetate (3:1, *v*/*v*) as an eluent to give product **2** as a colorless solid. The ee value was determined by HPLC using a chiral stationary phase.

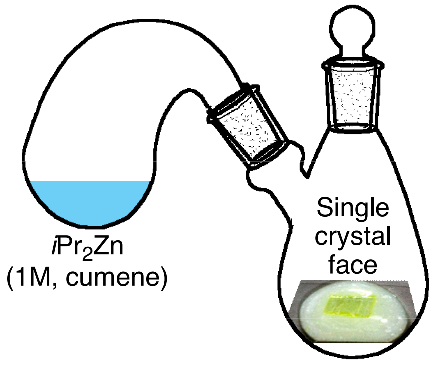

Figure S1. Reaction apparatus for the addition of *i*Pr_2Zn vapor to the single enantiotopic surface of the achiral crystal of achiral aldehyde **1**.

Data for (*S*)-alkanol 2 (> 99.5% ee): Colorless crystal; mp. 114.7–115.5 °C; $[\alpha]_D^{23}$ –14.6 (c 1.0, $CHCl_3$); ^{1}H-NMR (600 MHz, $CDCl_3$) δ (ppm) 0.227 (6H, s), 0.880 (3H, d, *J*=6.6 Hz), 0.949 (3H, d, *J*=7.2 Hz), 1.015 (9H, s), 1.975 (1H, dhept, *J*=6.6, 6.6 Hz), 2.176 (1H, br), 4.523 (1H, d, *J*=5.4 Hz), 8.643 (2H, s); ^{13}C-NMR (600 MHz, $CDCl_3$) δ (ppm) –4.67, 16.89, 17.53, 18.58, 26.34 35.45,92.9, 103.3, 135.01, 151.66, 155.84; IR (nujol) ν (cm^{-1}) 3370, 2955, 2929, 2854, 1416, 1252, 1054, 869, 845, 779; HRMS Calcd. for $C_{16}H_{26}N_2SiONa^+$ [M+Na]$^+$: 313.1707, found. 313.1712; HPLC (Figure S2):

Daicel Chiralpak IB column (250 x 4.6 Φ mm ID), eluent: 3% 2-propanol in hexane (*v*/*v*), flow rate 1.0 mL/min, 254 nm UV detector, retention time 10.4 min for (*S*)-**2**, 12.7 min for (*R*)-**2**.

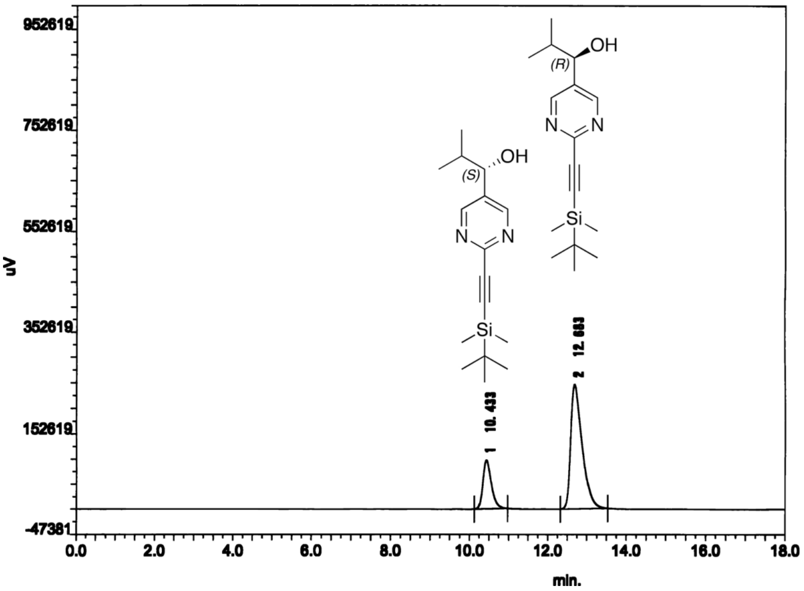

Figure S2. HPLC spectrum of (*R*)-alkanol **2** with 55% ee (Table 1, entry 9).

3. Asymmetric autocatalysis of alkanol2 with amplification of ee (Table S1):

To a solution of alkanol **2** (2.9 mg, 0.01 mmol) in the solvent (4 mL), 1 M diisopropylzinc solution (0.22 mL, 0.22 mmol) was added at 0 °C. After stirring 15 min, aldehyde **1** (24.6 mg, 0.1 mmol) in the solvent (2 mL) was added dropwise for a period of 1 h. After the mixture was stirred for 2 h at 0 °C, the reaction was quenched with saturated aqueous NH_4Cl. The mixture was extracted using ethyl acetate three times. The combined organic layers were dried over anhydrous sodium sulfate and evaporated in vacuo. Purification of the residue using silica gel column chromatography (hexane/ethyl acetate = 3/1, v/v) gave alkanol 2. The ee was determined using HPLC employing a chiral stationary phase.

Table S1. Consecutive asymmetric autocatalysis of (*R*) and (*S*)-pyrimidyl alkanol **2** with significant amplification of enantiomeric purity.

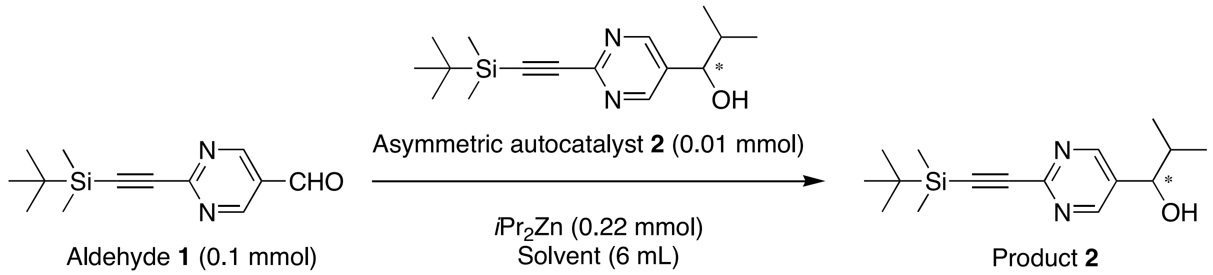

entry[a]	ee of autocatalyst **2** (% ee)	newly formed product **2**	newly formed product and autocatalyst **2**	Solvent
		yield[b] (%)	ee (% ee)	
1[c]	44 (*R*)	90	90 (*R*)	Cumene
2	90 (*R*)	88	98 (*R*)	Cumene
3	98 (*R*)	87	>99.5 (*R*)	Et_2O
4[d]	31 (*S*)	79	89 (*S*)	Cumene
5	89 (*S*)	91	98 (*S*)	Cumene
6	98 (*S*)	85	>99.5 (*S*)	Et_2O

[a] Unless otherwise noted, the molar ratio used was asymmetric autocatalyst **2**:pyrimidine-5-carbaldehyde **1**:$i$$Pr_2Zn$ = 0.01:0.1:0.22.

[b] The yield was calculated by subtracting the amount of asymmetric autocatalyst **2** loading from the obtained mixture.

[c] Alkanol **2** (0.0075 mmol) was used as asymmetric autocatalyst.

[d] Alkanol **2** (0.0063 mmol) was used as asymmetric autocatalyst.
